# Prevalence of intestinal parasites in street dogs (*Canis lupus familiaris*) with highlights on zoonosis in Lalitpur, Nepal

**DOI:** 10.1002/vms3.1258

**Published:** 2023-09-05

**Authors:** Roshan Babu Adhikari, Madhuri Adhikari Dhakal, Tirth Raj Ghimire

**Affiliations:** ^1^ Nepali Army College of Health Sciences (NACHS) Kathmandu Nepal; ^2^ Third Pole Conservancy Bhaktapur Nepal; ^3^ Institute of Medical Science Alka Hospital Pvt. Ltd. Lalitpur Nepal; ^4^ Nepal Academy of Science and Technology Lalitpur Nepal; ^5^ Department of Microbiology and Research and Development New Edge Microbials Albury New South Wales Australia; ^6^ Department of Zoology Tri‐Chandra Multiple Campus Tribhuvan University Kathmandu Nepal

**Keywords:** *Ancylostoma*, intestinal parasites, public health, street dogs, zoonosis

## Abstract

**Background:**

The presence of intestinal parasites influences the growth and well‐being of canids. Additionally, infected dogs and their faeces with considerably higher eggs/oocysts released per gram (epg/opg) of zoonotic parasites contribute to parasitic spillover to humans, domestic animals and sympatric wildlife.

**Objectives:**

The current study aimed to reveal the prevalence of intestinal parasites (protozoa and helminths) and to list the zoonotically significant parasites in free‐roaming street dogs in Lalitpur Metropolitan City, Nepal.

**Methods:**

Fresh faecal samples (*n* = 332) were collected from feral dogs of varying ages and sexes and transported to the research laboratory. The copro microscopic examination was carried out via direct wet mount, formalin ethyl acetate sedimentation, saturated salt flotation, acid‐fast staining and sporulation techniques.

**Results:**

Coproscopy revealed an overall 95.7% (318/332) prevalence rate with 23 diverse species of intestinal parasites (10 protozoa and 13 helminths). Among them, 5 protozoa and 11 helminths possessed zoonotic potential, and their overall prevalence was 92.5%. Helminth's overall prevalence was double that of the protozoa (87.7% vs. 43%). Polyparasitism was dominant over mono parasitism (79.5% vs. 16.3%), and co‐infection of up to seven species of parasites at a time was recorded.

**Conclusions:**

Urban street dogs harboured a higher prevalence of intestinal parasites that varied with age and sex. Since most of the reported parasites are zoonotic, dog density and parasitic richness indicate a greater spillover risk to humans and domestic animals. Furthermore, this study also provides appropriate ‘baseline’ data for assessing effective control measures against parasitic infestations among street dogs and controlling their transmission to humans.

## Graphical abstract

Open defecation by dogs may be risk factor for zoonotic parasitosis.

▪ Urban street dogs have a high prevalence of intestinal parasites with greater diversity.

▪ Open defecation by dogs at the human inhabitant area is risk of zoonosis.

▪ Antihelminthic treatment to urban dogs should be conducted.

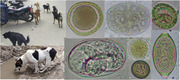

## 1 INTRODUCTION

Dogs (*Canis lupus familiaris*) (Family: Canidae), with the taxonomic serial number 726821 (www.itis.gov), are among the most ubiquitous and familiar domestic mammals. These canids were the first domesticated animals since prehistoric times (Freedman & Wayne, 2017) and are specially reared for social purposes like hunting companions, herders, protectors and friends by the human (Vanacore, 2022). In Nepal, dogs are important for Hindus who worship them; however, very few people raise them as pets, and most of them are freely living in the human habitat. In rural areas, they are fed by the community, but in urban centres, they aggregate around slaughterhouses, garbage piles, public places, temples and gumbas for their diet. Such free‐living canids are predominantly found in the Kathmandu valley and other urban centres in Nepal. Notably, due to the lack of effective deworming, immunization and supplement of adequate feed and shelter, they suffer from malnutrition and diseases (Massei et al., 2017). Such diseases occurring in the intestinal tract involve ancylostomiasis, capillariosis, coccidiosis, giardiasis, spirocercosis, tapeworm infection, toxocariosis, trichuriasis and others. They can induce minor to significant pathogenic effects, including severe morbidity and mortality (Kagira & Kanyari, 2000; Morelli et al., 2021; Schantz, 2007).

Although intestinal infections caused by gastrointestinal (GI) parasites lead to severe effects in dogs, these animals can act as reservoirs, vectors or carriers of bacterial, viral and parasitic agents and pose a threat or negative impact on humans as well as livestock and wildlife (Adhikari, Shrestha, et al., 2020; Fong, 2017; Ghasemzadeh & Namazi, 2015; Traub et al., 2002). Regarding this, several studies have explained the critical role of dogs in the emergence (or re‐emergence) of zoonosis (Baneth et al., 2016; Chomel, 2014; Ghasemzadeh & Namazi, 2015; Otranto et al., 2021) signifying, people with chronic illness, immunodeficiency and pregnancy are at the topmost risk. Human acquisition of parasite‐borne zoonotic diseases follows direct contact with infected dogs or exposure to the environment, food or water contaminated with dog's faeces (Schantz, 2007). The zoonotic parasites have been predominantly found with the prevalence rates ranging from 44.3% to 100% in feral dog population from global geographies, like India, China, Morocco, Nigeria and Uruguay, especially in the rural setting (Ayinmode et al., 2016; Fang et al., 2015; Idrissi et al., 2022; Malgor et al., 1996; Traub et al., 2002). On the other hand, 15% of clinical cases and 93% sero‐prevalence of canine‐specific *Ancylostoma* spp. (Heukelbach et al., 2008) and *Toxocara canis* (Magnaval et al., 1994) have been reported from Brazil and La Reunion, respectively. Similarly, several cases of canine‐specific whipworm (*Trichuris vulpis*) infection in humans have also been reported from endemic regions like Mexico (Márquez‐Navarro et al., 2012), India (Mirdha et al., 1998) and Thailand (Areekul et al., 2010). In these scenarios, external factors like environment and climate alternation, urbanization and habitat fragmentation might be important in parasite survival and transmission. In addition, the predatory pressure on small prey (rodents, birds and reptiles) might be the contributing factor to the acquisition as well as spillover of zoonotic parasites from feral canids (Mendoza Roldan & Otranto, 2023; Wells et al., 2018). In Nepal, a few helminthic faunae, like *Ancylostoma* sp., *Taenia* sp., *T. canis*, *Dipylidium caninum, Capillaria* sp. and *T. vulpis*, were recorded in the faecal samples of dogs with higher prevalence ranging from 46.7% to 70% (Sukupayo & Tamang, 2023; Yadav & Shrestha, 2017) and concluded that free‐ranging canids had a higher prevalence of zoonotic helminth parasites compared to the pet (Satyal et al., 2013). However, none of these native reports have discussed the presence of canine‐specific protozoan fauna. In addition, these authors have not discussed the presence of intestinal parasites with respect to zoonotic potential. Thus, to fulfil the knowledge gaps, in the current study, we selected free‐ranging canid populations from a recently urbanized area in Lalitpur district and aimed to update the information regarding prevalence of different endoparasites, including both protozoa and helminths in the openly defecated faecal samples. In addition, we aimed to explain how these parasites are distributed in different ages and sexes of the dogs and how these hosts can be sources of zoonotic parasitosis to the nearby domestic animals and humans.

## 2 MATERIALS AND METHODS

### 2.1 Study area

**FIGURE 1:**
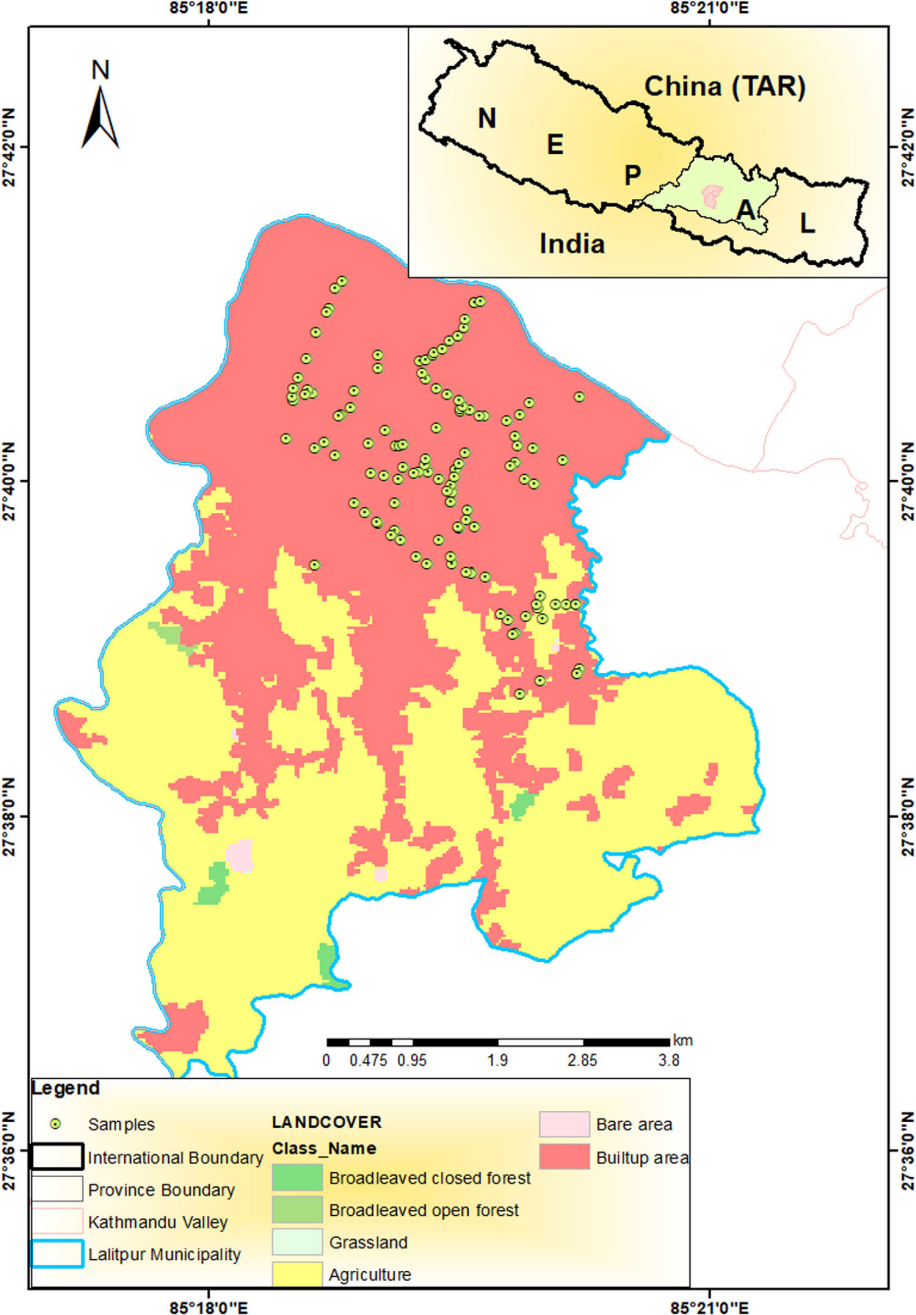
Map of study area.

The study was conducted in Lalitpur Metropolitan Municipality (27°32′53.88″ North, longitude 85°20′15.00″ East), the third largest city within the country (Figure 1) with higher rate of urbanization in recent years. It is situated in the south‐central part of the Kathmandu Valley, the country's capital in central Nepal. The city is well known for its rich cultural importance, particularly the ancient tradition, arts and crafts. The ancient monument ‘Patan Durbar Square’ listed by UNESCO as one of the World Heritage Site lies in this region. Lalitpur has a humid subtropical type of climate. The temperature is relatively high, with the highest annual range of 18–29°C and the lowest range of (3–20°C), even though it receives fairly distributed rainfall throughout the year ranging from 153 to 241 mm (Retrieved on 3 February 2022 from http://hikersbay.com/climate‐conditions/nepal/patan/climate‐conditions‐in‐lalitpur.html?lang = en).

**FIGURE 2:**
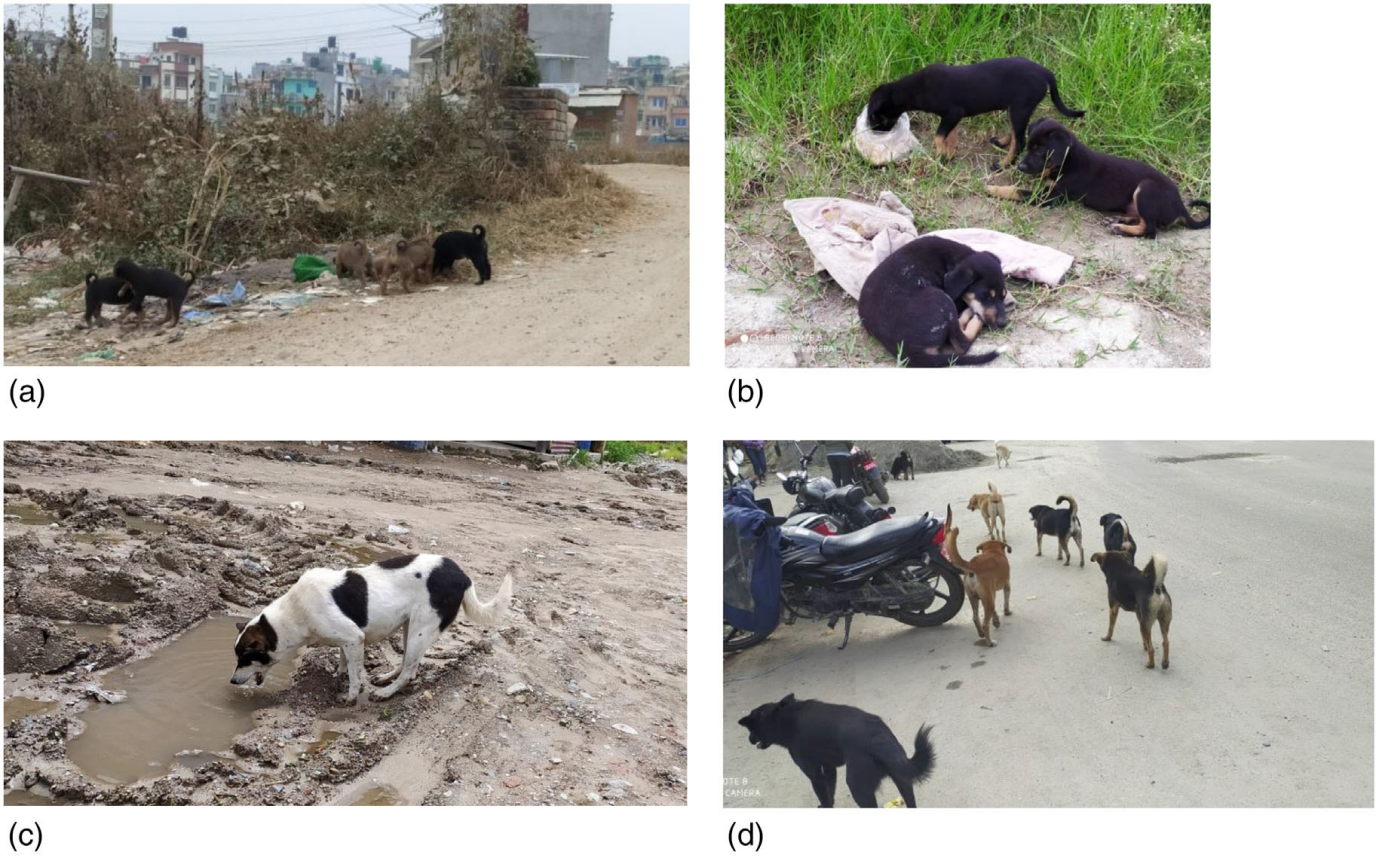
Feral dogs in human inhabitant areas. (A and B) Puppies in the garbage. (C) An adult licking water. (D) Feral dogs freely roaming.

According to Dog Survey Report 2015, 2793 dogs live freely on the streets of Lalitpur Metropolitan City (https://animalnepal.wordpress.com/2016/01/26/animal‐nepal‐launches‐lalitpur‐dog‐population‐survey‐report/), and this population is even more in today's date. These dogs roam freely in the streets, and their population seems to be highly concentrated towards slaughterhouses, religious sites, butcher shops and dumping sites where they scavenge for food (Figure 2A–D).

### 2.2 Sample collection, preservation and transportation

A total of 332 fresh faecal samples from 332 street dogs of varying age and sex groups were non‐invasively collected from 51 different locations within the metropolitan city. Since determining the age of free‐ranging canids is a challenging task, we simply categorized them as ‘young’ and ‘adult’ based on the differences in their morphology, adopted behaviour and the way of living. Younger ones are more playful and mischievous in nature. Morphologically, they have smaller stature, larger paws, and excess and loose skin and usually live in association with their mother. In contrast to this, adults are physically larger, with greyish and elongated muzzles, and have independent ways of life. On other hand, the sexes of these dogs were confirmed via direct observation of their genitals or via observing their sexually dimorphic urinary behaviour. Male generally lift their one hind leg whereas females squat while urinating (Wirant & McGuire, 2004). Faecal collection was performed on a daily basis early in the morning. For this, the free‐ranging canine troops were followed until they defecated and the portion of sample that had not touched the ground was carefully picked up using a metallic spatula and gloved hands. Initially, the faecal samples were macroscopically examined for faecal consistency and the presence of blood, mucus, segments of cestodes and dead nematodes and then kept in screw‐capped 20‐mL sterile vials. The samples were further added to 2.5% weight/volume (w/v) potassium dichromate solution and transported to the Animal Research Laboratory at Nepal Academy of Science and Technology for microscopic examination and parasitic investigation. Before the investigation, the samples were stored in the refrigerator at 4°C temperature for 2 weeks.

### 2.3 Laboratory processing and examination

The faecal samples were microscopically examined by four different techniques, including direct wet mount, formalin‐ethyl acetate (FEA) sedimentation, saturated salt (45% w/v NaCl) flotation and acid‐fast method based on the procedure previously described (Adhikari & Ghimire, 2021; Adhikari, Adhikari Dhakal, et al., 2021; Adhikari, Ale, et al., 2022; Adhikari, Dhakal, et al., 2022; Adhikari, Maharjan, et al., 2020; Adhikari, Parajuli, et al., 2021; Aryal et al., 2021). Furthermore, sporulation assays (Ghimire et al., 2021) were also performed to identify coccidian parasites like *Cystoisospora*, *Sarcocystis* and *Eimeria*.

#### 2.3.1 Direct wet mount technique

A sample drop mixed in 2.5% weight/volume (w/v) potassium dichromate solution was placed on a glass slide and observed under the microscope (40×).

#### 2.3.2 Concentration technique

Concentration techniques were followed by FEA sedimentation and saturated salt floatation methods. For the former process, 2 g of the sample was subsequently centrifuged (1200 rpm × 5 min) initially with normal saline (0.9% w/v NaCl) and then with a mixture of 10 mL of 10% formalin and 4 mL of ethyl acetate. A single drop of the final sediment was then placed on a glass slide and observed under the microscope (10×, 40×). For the saturated salt flotation method, the sediments obtained after the first centrifugation were added to 12 mL of 45% w/v of sodium chloride (NaCl) solution and then followed by centrifugation (1200 rpm × 5 min). Then, the tube was allowed to stand vertically on the test tube stand, and the flotation media was added drop by drop to fill it. The mouth of the tube was covered with a coverslip and left undisturbed for 10 min. At last, the coverslip was recovered and placed on the glass side for microscopic observation (40×).

#### 2.3.3 Acid‐fast staining technique

*Cryptosporidium‐* and *Cyclospora‐*positive sediments observed in FEA sedimentation were used to prepare a thin smear. The smear was initially fixed in absolute methanol for 2 min, then it was counter‐stained with carbol fuchsin for 15 min. Following it, the smear was de‐stained with acid alcohol for a while and then subsequently stained with malachite green for a minute. Finally, the slide was allowed for drying, and using immersion oil, the parasite was observed under the microscope (100×).

#### 2.3.4 Sporulation assays

About 5 g of each coccidia (*Eimeria, Cystoisospora* and *Sarcocystis*) positive sample was poured into a separate Petri dish. It was then mixed with 2.5% potassium dichromate solution in the ratio 1:5 and then incubated at 28°C for a week in an incubator. In every 24 h’ interval, the sporulation state of the oocysts was checked following the flotation technique.

#### 2.3.5 Measurement of parasitic burden/severity of infection

A 2 Cell McMaster Counting Slide (Hawksley and Sons Ltd) was used to measure the burden of parasite infection. It was measured by quantifying the number of eggs/oocysts of parasites released per gram of faeces (epg/opg). The procedure was followed with the instruction of the manufacturer's company and previously documented literature (Adhikari, Adhikari Dhakal, et al., 2021; Soulsby, 2012).

### 2.4 Parasite identification

All faecal samples were observed under a compound microscope (Optika Microscopes, B‐383PLi) (Magnification: 10×, 40×, and 100× of the objective lens) (Adhikari, Maharjan, et al., 2020). Photographs of the detected parasitic bodies (eggs/cysts/oocysts/trophozoites/larvae) were captured by the camera (SXView 2.2.0.172 Beta (6 November 2014, Copyright (C) 2013–2014) accompanied by the microscope (Adhikari, Maharjan, et al., 2020). The ImageJ 1.51k (National Institute of Health) was used for micrometry of the parasites. Their identification was performed based on previously illustrated literature (Soulsby, 2012; Zajac et al., 2021).

### 2.5 Data analysis

The data obtained were expressed as the number of positive samples and prevalence rates in the table using Microsoft Word 2010. Prevalence rates were calculated by dividing the number of GI‐positive samples (total or particular species) by the total number of samples observed and multiplied by 100. We used GraphPad Software (Prism 5 for Windows, Version 5.00@1992–2007, GraphPad Software, Inc) to analyse the prevalence rates between different variables like age and sex of dogs by applying Fisher's exact test (two‐sided) to assess *p‐*values. Moreover, statistical significance was considered at the 95% confidence interval (*p* < 0.05).

## 3 RESULTS

In the current study, 318 (95.7%) out of 332 faecal samples of street dogs were positive for intestinal parasites, and 307 samples (92.5%) had at least one species of zoonotic parasite. Altogether, 23 diverse species of parasites were reported (Table 1). Zoonotically significant parasites included five protozoa (*Cryptosporidium* sp., *Entamoeba* sp., *Giardia* sp., *Sarcocystis* spp. and *Balantidium coli*) and 11 helminths (*Ancylostoma caninum, Ancylostoma braziliense*, Taeniid, *T. canis*, *T. vulpis, Strongyloides* sp., *D. caninum*, Strongyle, *Troglotrema salmincola*, *Capillaria plica* and *Capillaria aerophila*) (Table 1) (Figure 3a‐v). The faecal consistency followed the order; hard and constipated stool (2.4%), formed stool (77.5%), loose stool (16.3%) and watery diarrhoea stool (3.8%); and except 14 formed consistency stools, all were positive for GI parasites.

**TABLE 1:** Age and sex‐wise prevalence of zoonotic and non‐zoonotic parasites in urban street dogs (N = 332).

	Young (*n*a = 140)		Adults (*nb* = 192)		Overall (N = 332)		
GI parasites	Male (*n*1 = 80)	Female (*n*2 = 60)	Male (*n*3 = 107)	Female (*n*4 = 85)	Prevalence (%)	*p‐*Values (age‐wise)	*p‐*Values (sex‐wise)
*Zoonotic protozoa*							
*Cryptosporidium* sp.	16 (20%)	8 (13.3%)	8 (7.5%)	9 (10.6%)	41 (12.3%)	ns	ns
*Entamoeba* sp.	17 (21.3%)	11 (18.3%)	6 (5.6%)	8 (9.4%)	42 (12.7%)	0.011	ns
*Giardia* sp.	12 (15%)	8 (13.3%)	8 (7.5%)	5 (5.9%)	33 (9.9%)	ns	ns
*Sarcocystis* spp.	5 (6.3%)	3 (5%)	10 (9.3%)	6 (7.1%)	24 (7.2%)	ns	ns
*Balantidium coli*	1 (1.3%)	0 (0%)	2 (1.9%)	1 (1.2%)	4 (1.2%)	ns	ns
*Total*	39 (48.6%)	31 (51.7%)	39 (36.4%)	28 (32.9%)	137 (41.3%)	ns	ns
**Zoonotic helminths**							
*Ancylostoma caninum*	34 (42.5%)	21 (35%)	57 (53.3%)	44 (51.8%)	156 (47%)	0.011	ns
*Ancylostoma braziliense*	12 (15%)	9 (15%)	32 (30%)	27 (31.8%)	80 (24.1%)	0.015	ns
Taeniid	11 (13.8%)	8 (13.3%	31 (29%)	24 (28.2%)	74 (22.3%)	ns	ns
*Toxocara canis*	15 (18.8%)	18 (30%)	24 (22.4%)	14 (16.5%)	71 (21.4%)	ns	ns
*Trichuris vulpis*	17 (21.3%)	11 (18.3%)	21 (19.7%)	21 (24.7%)	70 (21.1%)	ns	ns
*Strongyloides* sp.	8 (10%)	9 (15%)	19 (17.8%)	10 (11.8%)	46 (13.9%)	0.040	ns
*Dipylidium caninum*	6 (7.5%)	3 (5%)	20 (18.7%)	10 (11.8%)	39 (11.7%)	ns	ns
Strongyle	7 (8.8%)	0 (0%)	17 (15.9%)	7 (8.2%)	31 (9.3%)	0.028	ns
*Troglotrema salmincola*	3 (3.8%)	1 (1.6%)	9 (8.4%)	13 (15.3%)	26 (7.8%)	ns	ns
*Capillaria plica*	4 (5%)	3 (5%)	2 (1.9%)	3 (3.5%)	12 (3.6%)	ns	ns
*Capillaria aerophila*	2 (2.5%)	1 (1.7%)	2 (1.9%)	3 (3.5%)	8 (2.4%)	ns	ns
Total	65 (81.3%)	49 (81.7%)	94 (87.9%)	77 (90.6%)	285 (85.8%)	ns	ns
**Non zoonotic protozoa**							
*Cystoisospora canis*	12 (15%)	14 (23.3%)	12 (11.2%)	12 (14.1%)	50 (15.1%)	ns	ns
*Cystoisospora ohioensis*	6 (7.5%)	11 (18.3%)	15 (14%)	7 (8.2%)	39 (11.7%)	ns	ns
*Eimeria* spp.	2 (2.5%)	1 (1.7%)	2 (1.9%)	3 (3.5%)	8 (2.4%)	0.028	ns
*Hammondia/Neospora*	3 (3.8%)	0 (0%)	2 (1.9%)	5 (5.9%)	10 (3%)	ns	ns
**Non‐zoonotic helminth**							
*Spirocerca lupi*	0 (0%)	0 (0%)	7 (6.5%)	4 (4.7%)	11 (3.3%)	ns	ns
**Zoonotic potential unknown species**							
*Cyclospora* sp.	0 (0%)	1 (1.7%)	1 (0.9%)	0 (0%)	2 (0.6%)	ns	ns
*Toxascaris leonina*	0 (0%)	0 (0%)	8 (7.5%)	4 (4.7%)	12 (3.6%)	ns	ns
**Total protozoa**	39 (48.8%)	33 (55%)	42 (39.3%)	29 (34.1%)	143 (43.1%)	0.011	ns
**Total helminths**	67 (83.8%)	50 (83.3%)	95 (88.8%)	79 (92.9%)	291 (87.7%)	ns	0.0165
**Grand total**	78 (97.5%)	58 (96.7%)	99 (92.5%)	83 (97.6%)	318 (95.7%)	ns	ns

*Note*: *p*‐Values were assessed by Fisher's exact tests.

Abbreviation: GI, gastrointestinal.

FIGURE 3Gastrointestinal parasites identified in the urban street dogs. (a) Cyst of *Entamoeba* sp. (10 × 10 μm), 400×, after direct wet mount technique. (b) Oocysts of *Cryptosporidium* sp. (6 × 5 μm), 1000× after acid‐fast staining technique. (c) Oocyst of *Cyclospora* sp. (9 × 9 μm), 400× after direct wet mount technique. (d) Oocyst of *Hammondia/Neospora* (13 × 12 μm), 400× after flotation technique. (e) Oocyst of *Cystoisospora ohioensis* (26 × 24 μm), 400× after flotation technique. (f) Oocyst of *Cystoisospora canis* (42 × 34 μm), 400× after flotation technique. (g) Oocyst of *Eimeria* sp. (25 × 17 μm), 400× after flotation technique. (h) Oocysts of *Sarcocystis* spp. (13 × 10 and 16 × 13 μm), 400× after flotation technique. (i) Eggs of Taeniid (24 × 22 and 33 × 32 μm), 400× after FEA sedimentation technique. (j). Egg of *Spirocera lupi* (35 × 18 μm), 400× after sedimentation technique. (k) Egg of *Strongyloides* sp. (56 × 35 μm), 400× after flotation technique. (l) Cyst of *B. coli* (54 × 52 μm), 400× after direct wet mount technique. (m) Egg of *Ancylostoma braziliensis* (67 × 37 μm), 400× after flotation technique. (n) Eggs of Strongyle 1 (89 × 46 μm), 400× after flotation technique. (o) Eggs of Strongyle 2 (80 × 40 μm) and *Ancylostoma caninum* (55 × 34 μm), 400× after flotation technique. (p) Egg of Strongyle 3 (111 × 37 μm), 400× after direct wet mount technique. (q) Egg of *Toxocara canis* (67 × 64 μm), 400× after FEA sedimentation technique. (r) Egg of *Dipylidium caninum* (149 × 115 μm), 400× after FEA sedimentation technique. (s) Egg of *Troglotrema salmincola* (62 × 31 μm), 400× after FEA sedimentation technique. (t) Egg of *Trichuris vulpis* (82 × 41 μm), 400× after FEA sedimentation technique. (u) Egg of *Capillaria aerophila* (80 × 36 μm), after FEA sedimentation technique. (v) Egg of *Capillaria plica* (58 × 26 μm), 400× after FEA sedimentation technique.

**Figure:**
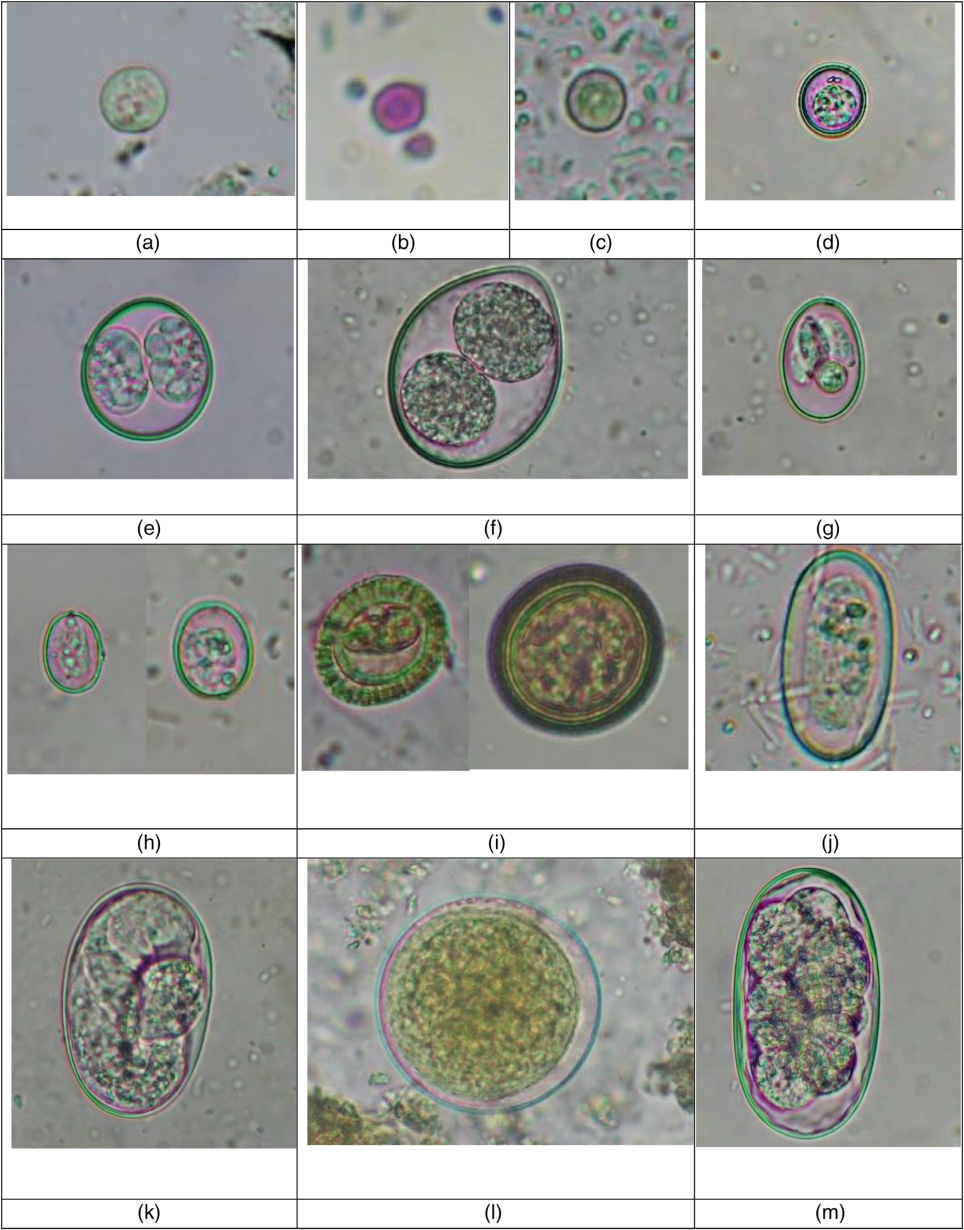

**Figure:**
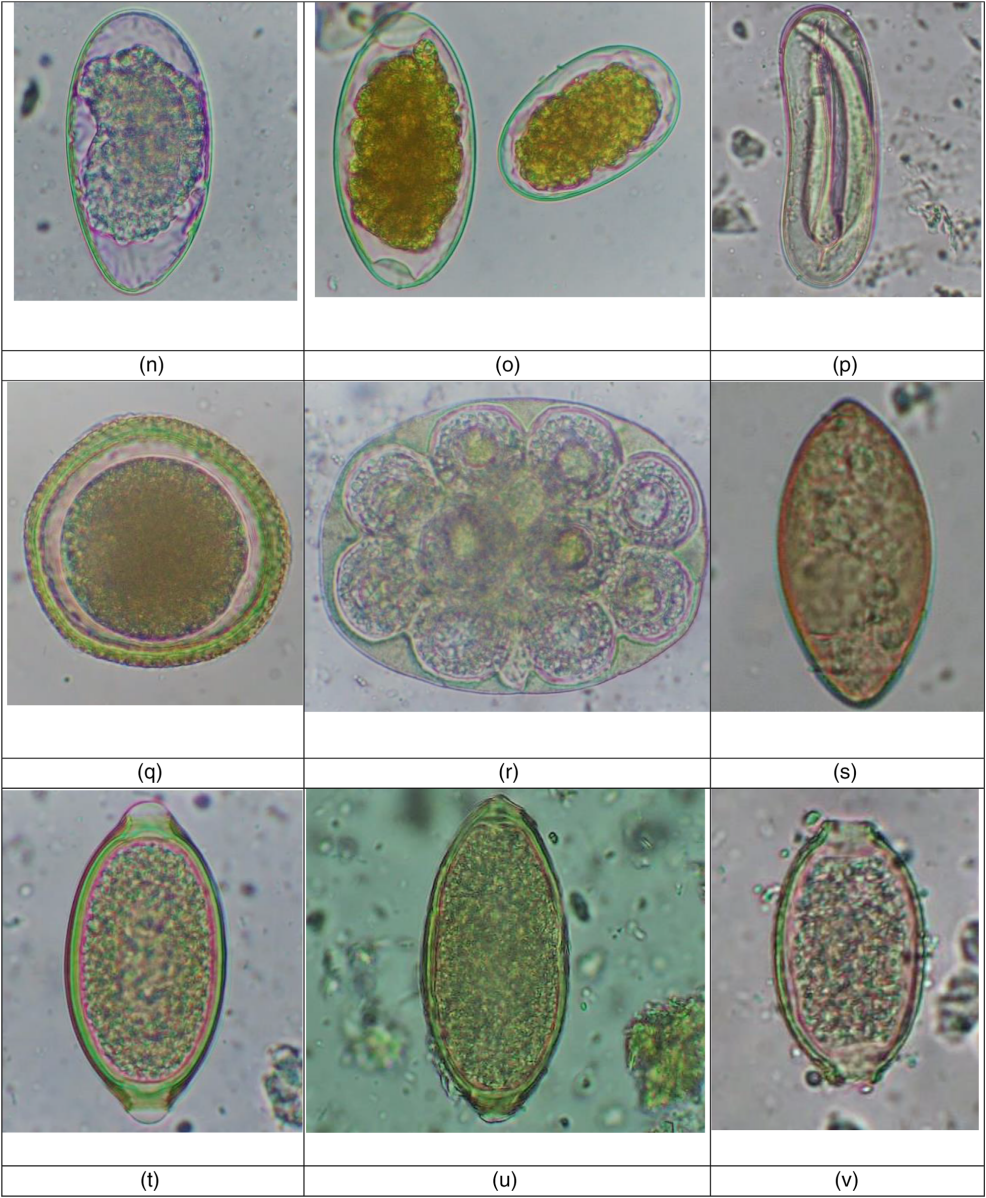

The young dogs (*n*1 = 140) were infected with 21 species while the adults (*n*2 = 192) were infected with 23 species of intestinal parasites. However, both young and adults were infected with 16 zoonotic species. *A. caninum* and *T. canis* were most common among the young, whereas *A. caninum*, *A. braziliense* and Taeniid were highly prevalent among the adults. Age‐wise statistical significant difference was observed in the context of the prevalence of total protozoa as well as *Entamoeba* sp., *A. caninum, A. braziliense*, *Strongyloides* sp., Strongyle and *Eimeria* spp. (*p* < 0.05).

Furthermore, female dogs had a slightly higher prevalence of intestinal parasites (97.2% vs. 94.7%) and zoonotic species of parasites (89.1% vs. 81.4%) than males. Young males were highly infected with *A. caninum* and *Entamoeba* sp., whereas young females were highly infected with *A. caninum* and *T. canis*. Both adult males and females were dominantly infected by *Ancylostoma* spp. (*A*. *caninum* and *A*. *braziliense*). Gender of dogs was significantly associated with the prevalence of total helminths only (*p* < 0.05), although variations in the data were observed.

In this study, polyparasitism was dominant over monoparasitism (79.5% vs. 16.3%) irrespective of age and sex (Table 2). The highest concurrency of up to seven species of parasites was recorded. Young dogs had concurrency with two to five species of parasites, whereas adult dogs were co‐infected with two to seven species of parasites. Age‐wise infections of parasitic species up to five species were statistically significant (*p* < 0.05), although sex‐wise statistical variations were not present (*p* > 0.05). One of the unique findings was the oocyst similar to *Hammondia heydorni* or *Neospora caninum*, which we named ‘*Hammondia/Neospora’* based on similar oocyst morphology (Slapeta et al., 2002). Furthermore, assessment of parasitic burden revealed that current canids were shedding large numbers of eggs of helminths and oocysts of coccidian per gram (Table 3). Young canids were excreting a higher count of oocysts of *Cystoisospora* spp. and eggs of *Ancylostoma* spp., *T. canis* and *Strongyloides* sp., whereas the adults were discharging the higher number of eggs of Strongyle, *Trichuris* sp. and *Capillaria* spp. and oocysts of *Sarcocystis* spp. Conspicuously, young females and adult males were reported to release the highest epg/opg of most of the zoonotic parasites (Table 3).

**TABLE 2:** Pattern of co‐infection.

	Young (*n*1 = 140)		Adults (*n*2 = 192)		Overall (*n* = 332)	*p‐*Values (age‐wise)	*p‐*Values (sex‐wise)
GI parasites	Male (*n*1 = 80)	Female (*n*2 = 60)	Male (*n*3 = 107	Female (*n*4 = 85)	Prevalence (%)	ns	ns
**Single**	15 (18.6%)	12 (20%)	14 (13.1%)	11 (12.9%)	52 (15.7%)	ns	ns
**Double**	22 (27.5%)	16 (26.7%)	19 (17.8%)	13 (15.3%)	70 (21.1%)	ns	ns
**Triple**	30 (37.5%)	25 (41.7%)	31 (29%)	36 (42.4%)	122 (36.7%)	ns	ns
**Quadruple**	9 (11.3%)	6 (10%)	15 (14%)	12 (14.1%)	42 (12.7%)	ns	ns
**Pentuple**	1 (1.3%)	0 (0%)	12 (11.2%)	7 (8.2%)	20 (6%)	0.0097	ns
**Hexuple**	0 (0%)	0 (0%)	4 (3.7%)	3 (3.5%)	7 (2.1%)	ns	ns
**Septuple**	0 (0%)	0 (0%)	3 (2.8%)	0 (0%)	3 (0.9%)	ns	ns

*Note*: *p*‐Values were assessed by Fisher's exact tests.

Abbreviation: GI, gastrointestinal.

**TABLE 3:** Burden (eggs/oocysts per gram, epg/opg) in the faecal samples with respect to age and sex of the dogs.

	Average range of epg/opg				
	Young		Adults		
Parasites	Male	Female	Male	Female	Total
*Ancylostoma* spp.	200–8200	200–9200	300–8400	100–7200	200–9200
*Cystoisospora* spp.	400–10,500	200–12,500	100–4200	100–3900	100–12,500
*Sarcocystis* spp.	100–2200	200–2600	100–6500	100–5600	100–6500
*Toxocara canis*	400–16,000	500–16,500	200–12,500	100–8400	200–16,500
*Strongyloides* sp.	100–2000	100–1400	100–1200	100–800	100–2000
*Trichuris* sp.	100–1600	100–1800	100–1600	100–2200	100–2200
*Capillaria* spp.	100–400	100–500	100–700	100–400	100–700
Strongyle	100–700	100–500	100–1400	100–1100	100–1400

It was further important that we reported four morphotypes of Strongyles (size range: 67–111 × 33–46 μm), three morphotypes of *Eimeria* spp. (size range: 23–33 × 17–26 μm) and three morphotypes of *Sarcocystis* spp. (size range: 13–21 × 10–14 μm).

## 4 DISCUSSION

The current study first identified a considerable diversity of intestinal parasites from free‐ranging canids in the study area and Nepal. The current prevalence rate of intestinal parasites (95.7%; *n* = 332) was on par with the report of stray canids from Bangladesh (95%; *n* = 60) (Das et al., 2012), lower than from Ethiopia (100%; *n* = 13) (Jones et al., 2011) and India (99%; *n* = 101) (Traub et al., 2014) but was higher than from Nepal (56.2%–78.5%; *n* = 105–157) (Satyal et al., 2013; Yadav & Shrestha, 2017), India (90.7%; *n* = 108) (Sudan et al., 2015), South Africa (82.5%; *n* = 240) (Mukaratirwa & Singh, 2010), Malaysia (87.7%, *n* = 77 to 88.3%, *n* = 227) (Ngui et al., 2014; Tun et al., 2015) and Vietnam (55.5%, *n* = 200) (Ng‐Nguyen et al., 2015). It indicates that the prevalence rate of intestinal parasites is different in different countries; these variations might be due to the difference in geo‐climatic factors, sample size, sampling breed, sampling season, treatment strategy and methodological contrasts. In the current study, even though we did not consider the sampling breed, we also collected openly defecated faeces during seasons when the temperature, rainfall and humid climates favour the survival, development and transmission of various stages of the parasites like cysts, larvae and eggs (Drake & Carey, 2019; Igore et al., 2020). During rainy days, the faeces are swept away, are widespread around the human settlements and have a high potential to disseminate parasites. This can be zoonotically critical in some of the study sites, which have a dense human population. Therefore, the presence of openly defecating free‐ranging dogs in crowded areas, in the rainy season, might be a risk factor for parasitic zoonosis although further epidemiological proofs must warrant it.

The primary reason for the higher prevalence and greater diversity of intestinal parasites and zoonotic parasites in the present study might be the scavenging feeding habit of the canids. The feral canids are highly mobile; thus scavenge mostly nearby slaughterhouses, butcher shops, hotels, restaurants, temples and landfill sites. They feed on animal carcasses, leftover foods, garbage, animal dung and human faeces. Furthermore, they also lick water from contaminated sources that contribute to parasitosis. Although few organizations work for the welfare of street dogs in Nepal (https://myrepublica.nagariknetwork.com/news/kathmandu‐has‐23‐000‐street‐dogs/), vaccination, deworming and castration are not optimal; thus, they may result in enhanced susceptibility to parasitism (Chidumayo, 2018).

Furthermore, stray dogs are reservoirs of many intestinal parasites, including zoonotic ones (Trasviña‐Muñoz et al., 2020; Traub et al., 2014). Current research shows that the overall prevalence of enteric parasites and zoonotic species depends on the age of the canids. Similar results with greater prevalence of enteric parasites in young were obtained from previous studies from Nepal (Satyal et al., 2013; Yadav & Shrestha, 2017), and Ecuador (Sangache et al., 2020), whereas studies from Zambia (Mugala et al., 2018) and Mexico (Cantó et al., 2011; Trasviña‐Muñoz et al., 2020) reported contrasting results. Currently, zoonotic species like *Entamoeba* sp., *Giardia* sp*., Cryptosporidium* sp. and *T. canis* were predominant in the young, but *Ancylostoma* spp., Taeniid, *Strongyloides* sp., *D. caninum*, Strongyle, *T. salmincola*, *T. vulpis* and *Capillaria* spp. were dominant in the adults. It is not easy to explain this predilection. However, it has been discussed that due to naïve immune status, the newborn offspring can be infected by protozoa with high pressure (Barutzki & Schaper, 2011). A similar result has been obtained in the context of *T. canis* nematodes in young compared to adults (Nijsse et al., 2016). Prior infection by parasites in the young generates a protective immunological memory in adults and protects them from new infection (Jensen et al., 2015) resulting in reduced parasitic prevalence. Therefore, further studies are necessary to assess why the parasite‐specific predominance differs in different ages.

Regarding the sex, prevalence of overall enteric parasites in females had higher than males, although similar ranges of zoonotic parasites were detected in both sexes. These findings are in agreement with the previous reports from Nepal (Satyal et al., 2013), Mexico (Trasviña‐Muñoz et al., 2020) and Zambia (Mugala et al., 2018). However, other studies from Nepal (Yadav & Shrestha, 2017), Mexico (Cantó et al., 2011) and Ecuador (Sangache et al., 2020) completely disagreed with it. Physiologically, female canids have higher cortisol concentrations than males (Sundman et al., 2019). Thus, it is speculated that they may have higher stress, which might cause weak immunity and greater susceptibility to parasitic infections (Fleming, 1997; Wakelin, 1984). In addition, owing to the partially suppressed immune system at whelping, females may acquire a wide variety of parasites (Fleming, 1997; Pereira et al., 2019; Wakelin, 1984). Furthermore, unlike the female, males’ fitness increases with promiscuous mating behaviour (insemination to multiple females) (Scandurra et al., 2018; Shuster & Wade, 2019) which might decrease parasitic acceptance and establishment. However, further studies should confirm these hypotheses.

Noticeably, helminth parasites had a higher prevalence and a wider variety than protozoa. A higher prevalence of helminth parasites in stray dogs has already been reported in India (Sudan et al., 2015) and South Africa (Mukaratirwa & Singh, 2010). These regions exhibit subtropical to tropical climatic conditions. In this study, *Ancylostoma* spp. was the most prevalent parasite. This indicates that egg hatching and larval development of ancylostomatid worms are favoured by the subtropical climatic condition of the study areas. Hookworm induces threatening anaemia in canids and causes eosinophilic enteritis and larval migrans syndromes in humans (Croese et al., 1994; McCarthy & Moore, 2000). Next to the hookworm was the Taeniid. The Taeniid is transmitted via scavenging on the carcasses of infected domestic herbivores. Although *Echinococcus granulosus*, *Echinococcus multilocularis*, *Taenia ovis*, *Taenia multiceps* and *Taenia hydatigena* are reported from canids, only, *E*. *granulosus* and *E. multilocularis* possess significant medical and public health risks (Cardona & Carmena, 2013; Eckert & Deplazes, 2004) suggesting a need of further molecular diagnosis. *D. caninum* is another zoonotic helminth that rarely infects humans; however, the zoonotic risk increases with the availability of a high proportion of canids excreting eggs to the environment (Gutema et al., 2021). Interestingly, *T. canis* can easily cross the host barrier and infect a wide range of hosts like cats and wild canids, including humans (Macpherson, 2013). With increasing cases of visceral toxocariasis, ocular toxocariasis, common toxocariasis and covert toxocariasis in humans from different geography (Magnaval et al., 1994; Magnaval et al., 2001; Singh et al., 2007; Chen et al., 2018), *T. canis* has been a concern for public health. However, the zoonotic potential of the currently detected canid *Toxascaris leonina* is still questionable. However, a case of ocular involvement in a child from East Africa had been reported (Beaver & Bowman, 1984). In addition, its larvae have been shown to invade the tissues of some laboratory animals (Despommier, 2003). Uniquely, this study first reported *T. salmincola* (*Nanophyetus salmincola*) in Nepalese dogs. The canids acquire their infection upon the ingestion of raw or undercooked flesh of fish encysted with infective metacercaria larva (Lin et al., 2017). Although non‐pathogenic, it acts as a vector of *Neorickettsia helminthoeca*, or salmon poisoning disease, and can result in higher mortality in dogs (Fritsche et al., 1989; Lin et al., 2017). It is critical for public health because it may infect humans as well as cats, raccoons, foxes and a few species of birds (Eastburn et al., 1987; Fritsche et al., 1989). Furthermore, *C. plica* and *C. aerophila* detected in this study have a history of human infection (Cross 1992). In the same way, a few clinical cases have also been attributed to *T. vulpis* (Areekul et al., 2010; Dunn et al., 2002), indicating zoonotic transmission in endemic regions.

Furthermore, regarding the protozoa, coccidia were the most dominant parasitic species. Except for the common *Cystoisospora* spp., the presence of *Cryptosporidium* sp. and *Sarcocystis* spp. might be critical for both canids and sympatric hosts. Canine cryptosporidiosis is caused by *C. canis, Cryptosporidium parvum, Cryptosporidium muris* and *Cryptosporidium meleagridis*; moreover, a few of them are zoonotically significant (Abe et al., 2002; Jian et al., 2014; Lupo et al., 2008). Its risk of cross‐transmission usually increases within the large population size and close contact of canids with other animals and humans. Similarly, human cases of *Sarcocystis* have been reported before (Fayer et al., 2015). Several species like *Sarcocystis cruzi, Sarcocystis caninum, Sarcocystis ovicanis, Sarcocystis miescheriana, Sarcocystis bertrami, Sarcocystis fayeri, Sarcocystis hemionilatrantis* and *Sarcocystis svanai* (Dubey, 2003; Dubey et al., 2006; Soulsby, 2012) have already been reported from canines. Since canids are predators and scavenge for food, consumption of raw meat/tissues and exposure to tainted faeces contribute to *Sarcocystis*. Another coccidian parasite, *Hammondia/Neospora*, can be critical for sympatric animals because of its causal association with abortion in cattle and other vertebrates (Dubey, 2003; Dubey et al., 2006). Notably, the presence of *Entamoeba* sp. and *Giardia* sp. in the current dog populations indicates that zoonotic possibilities in the nearby humans and animals with these protozoa should be further elaborated. Interestingly, *B. coli* in the faecal samples indicated that dogs could be important sources of zoonosis or cross‐transmission for humans, although ciliates are unusual and rarely infect the canine population (Chalmers, 2014).

Relating to the parasitic burden, current canid populations were reported to shed a moderate‐ to‐ higher number of eggs and oocyst (epg/opg) per gram in their faeces. The higher epg/opg count for parasites like *Ancylostoma* spp., *T. canis*, *Trichuris* spn2. and *Cystoisospora* spp. in the present study corresponds with the finding results from various geographies like Nigeria (Sowemimo, 2009), South Africa (Mukaratirwa & Singh, 2010) and Portugal (Cardoso et al., 2014). This indicates that feral dogs are prone to high burden of parasitic infection, which can be a sign of their frail GI health and intensified transmission risk.

Concerning the concurrency of the parasitic infection, co‐infection with multiple species of parasites is higher than a single infection, and irrespective of age and sex, triplet infection was highly reported. Polyparasitism generally occurs when one species of co‐infected member facilitates the occurrence of another or when the host is exposed to common risk factors (Vaumourin et al., 2015). Furthermore, host characteristics/behaviour, host distribution and habitat use, the host's life history traits and the nature of the infecting parasites determine the occurrence of polyparasitism (Vaumourin et al., 2015). Although it may result in positive, negative or neutral interactions among the coinfecting members within the host (Hoarau et al., 2020), its synergistic effect can lead to enhanced virulence and disease severity. For example, mixed infections cause disease severity in humans (Ezeamama et al., 2008; Sokhna et al., 2004), cattle (Adhikari & Ghimire, 2021), cats (Adhikari, Dhakal, et al., 2022; Saravanan et al., 2016), pigs (Adhikari, Adhikari Dhakal, et al., 2021; Morgan et al., 1999) and marine mammals (Gibson et al., 2011). Therefore, with reference to dominant polyparasitism accompanied by a considerable level of epg/opg count, the current feral dog populations might have impaired GI health and are sources of zoonosis in the study areas.

A few limitations of the current study should be considered. The foremost is the methodological constraint, which involves smear assessment and examination of single‐spot faecal samples. Such methods might not be efficient enough to address the pre‐patent infections and the intermittent shedding of the oocysts and eggs. Furthermore, it can also underrate the quantification of epg/opg burden, prevalence rate and polyparasitism. The species‐level identification of eggs and oocysts of parasites would have been better with molecular methods. Second, it is the cross‐sectional nature of study, which might be inadequate to determine the precise reason behind the zoonosis observed in the study. Yet, we stringently assured the quality control during our field surveys and laboratory techniques that have produced the best results of canid GI parasites.

## 5 CONCLUSIONS AND RECOMMENDATIONS

The current study detailed the diversity of intestinal, protozoan and helminth parasites of free‐ranging urban street dogs significant for zoonosis. Due to their scavenging nature, these dogs can act as reservoirs and primary hosts of many parasites. They shed the cysts, oocysts and eggs of the intestinal parasites into the environment openly. As a result, they can be critical sources of zoonosis in humans. Thus, it is a matter of concern for veterinarians and public health workers to design a strategic plan for deworming and vaccination to maintain their positive health and to check the possible risk of canid‐borne zoonoses. Similarly, an integrated approach of ‘One Health’ involving clinical, veterinary, environmental, ecological and socio‐economical factors must be considered to evaluate the possibility of cross‐transmission/zoonosis. In addition, detailed histopathological and molecular studies will support the causal association of single or mixed infections in canids.

## AUTHOR CONTRIBUTIONS

*Conceptualization; field and laboratory survey; methodology; data analysis; and original draft preparation; reviewing and editing*: Roshan Babu Adhikari. *Methodology; data analysis; reviewing; and editing*: Madhuri Adhikari Dhakal. *Conceptualization; laboratory analysis; data analysis; writing; reviewing; editing; and supervision*: Tirth Raj Ghimire. All authors approved the final version of the manuscript.

## CONFLICT OF INTEREST STATEMENT

The authors declared no potential conflicts of interest concerning the research, authorship and/or publication of this article.

## FUNDING INFORMATION

The authors received no financial support for the research, authorship and/or publication of this article. Laboratory facilities were provided by the Nepal Academy of Science and Technology.

## CONSENT

No animals or people are identifiable within this publication. All animals were free‐ranging; therefore, additional informed consent for publication was not required.

## ETHICS STATEMENT

The required permission for collecting the faecal samples was issued by Lalitpur Metropolitan City and Lalitpur Metropolitan Veterinary Service (Lalitpur, Nepal) (Permission No. 2950/077/078). The study was conducted using the stool samples defecated at the surface, and no experimental infection in dogs was established during the work.

## Data Availability

All data generated in the research has been submitted in this article.

## References

[vms31258-bib-0001] Abe, N. , Kimata, I. , & Iseki, M. (2002). Identification of genotypes of *Cryptosporidium parvum* isolates from a patient and a dog in Japan. Journal of Veterinary Medical Science, 64, 165–168. 11913556 10.1292/jvms.64.165

[vms31258-bib-0002] Adhikari, R. , & Ghimire, T. (2021). A case study of multiple parasitisms in a calf buffalo (*Bubalus bubalis*). Agricultural Science Digest, 41, 237–241.

[vms31258-bib-0003] Adhikari, R. B. , Adhikari Dhakal, M. , Thapa, S. , & Ghimire, T. R. (2021). Gastrointestinal parasites of indigenous pigs (*Sus domesticus*) in south‐central Nepal. Veterinary Medicine and Science, 7, 1820–1830. 34021721 10.1002/vms3.536 PMC8464252

[vms31258-bib-0004] Adhikari, R. B. , Ale, P. B. , Dhakal, M. A. , & Ghimire, T. R. (2022). Prevalence and diversity of intestinal parasites in household and temple pigeons (*Columba livia*) in central Nepal. Veterinary Medicine and Science, 8(4), 1528–1538. 10.1002/vms3.792 35352510 PMC9297752

[vms31258-bib-0005] Adhikari, R. B. , Dhakal, M. A. , Ale, P. B. , Regmi, G. R. , & Ghimire, T. R. (2022). Survey on the prevalence of intestinal parasites in domestic cats (*Felis catus* Linnaeus, 1758) in central Nepal. Veterinary Medicine and Science, 9, 559–571. 36346533 10.1002/vms3.999 PMC10029910

[vms31258-bib-0006] Adhikari, R. B. , Maharjan, M. , & Ghimire, T. R. (2020). Prevalence of gastrointestinal parasites in the frugivorous and the insectivorous bats in Southcentral Nepal. Journal of Parasitology Research, 2020, 8880033. 33414955 10.1155/2020/8880033 PMC7752302

[vms31258-bib-0007] Adhikari, R. B. , Parajuli, R. P. , Maharjan, M. , & Ghimire, T. R. (2021). Prevalence and risk factors of gastrointestinal parasites in the Chepangs in Nepal. Annals of Parasitology, 67, 387–405. 34953115 10.17420/ap6703.353

[vms31258-bib-0008] Adhikari, R. B. , Shrestha, M. , Puri, G. , Regmi, G. R. , & Ghimire, T. R. (2020). Canine distemper virus (CDV): An emerging threat to Nepal's wildlife. Applied Science and Technology Annals, 1, 149–154.

[vms31258-bib-0009] Areekul, P. , Putaporntip, C. , Pattanawong, U. , Sitthicharoenchai, P. , & Jongwutiwes, S. (2010). *Trichuris vulpis* and *T. trichiura* infections among schoolchildren of a rural community in northwestern Thailand: The possible role of dogs in disease transmission. Asian Biomedicine, 4, 49–60.

[vms31258-bib-0010] Aryal, M. , Adhikari, R. B. , Kandel, P. , Ghimire, T. R. , Khadka, D. , Maharjan, J. , Gaire, K. P. , Shrestha, S. , Manandhar, K. D. , Kandel, R. C. , Poudel, R. C. , & Pandey, K. (2021). First report on the molecular detection of *Entamoeba bovis* from the endangered wild water buffalo (*Bubalus arnee*) in Nepal. Veterinary Medicine and Science, 8(2), 799–807. 10.1002/vms3.697 34919350 PMC8959252

[vms31258-bib-0011] Ayinmode, A. B. , Obebe, O. O. , & Olayemi, E. (2016). Prevalence of potentially zoonotic gastrointestinal parasites in canine faeces in Ibadan. Nigeria Ghana Medical Journal, 50, 201–206. 28579625 10.4314/gmj.v50i4.2 PMC5443670

[vms31258-bib-0012] Baneth, G. , Thamsborg, S. , Otranto, D. , Guillot, J. , Blaga, R. , Deplazes, P. , & Solano‐Gallego, L. (2016). Major parasitic zoonoses associated with dogs and cats in Europe. Journal of Comparative Pathology, 155, S54–S74. 26687277 10.1016/j.jcpa.2015.10.179

[vms31258-bib-0013] Barutzki, D. , & Schaper, R. (2011). Results of parasitological examinations of faecal samples from cats and dogs in Germany between 2003 and 2010. Parasitology Research, 109, 45–60. 10.1007/s00436-011-2402-8 21739375

[vms31258-bib-0014] Beaver, P. , & Bowman, D. (1984). Ascaridoid larva (Nematoda) from the eye of a child in Uganda. The American Journal of Tropical Medicine and Hygiene, 33, 1272–1274. 6507736 10.4269/ajtmh.1984.33.1272

[vms31258-bib-0015] Cantó, G. , García, M. , García, A. , Guerrero, M. , & Mosqueda, J. (2011). The prevalence and abundance of helminth parasites in stray dogs from the city of Queretaro in central Mexico. Journal of Helminthology, 85, 263–269. 20849669 10.1017/S0022149X10000544

[vms31258-bib-0016] Cardona, G. A. , & Carmena, D. (2013). A review of the global prevalence, molecular epidemiology and economics of cystic echinococcosis in production animals. Veterinary Parasitology, 192, 10–32. 23084536 10.1016/j.vetpar.2012.09.027

[vms31258-bib-0017] Cardoso, A. , Costa, I. , Figueiredo, C. , Castro, A. , & Conceição, M. (2014). The occurrence of zoonotic parasites in rural dog populations from northern Portugal. Journal of Helminthology, 88, 203–209. 23388655 10.1017/S0022149X13000047

[vms31258-bib-0018] Chalmers, R. M. (2014). *Balantidium coli* . In Microbiology of waterborne diseases: Microbiological aspects and risks (2nd ed., pp. 277–286). Elsevier.

[vms31258-bib-0019] Chen, J. , Liu, Q. , Liu, G.‐H. , Zheng, W.‐B. , Hong, S.‐J. , Sugiyama, H. , Zhu, X.‐Q. , & Elsheikha, H. M. (2018). Toxocariasis: A silent threat with a progressive public health impact. Infectious Diseases of Poverty, 7, 1–13. 29895324 10.1186/s40249-018-0437-0 PMC5998503

[vms31258-bib-0020] Chidumayo, N. N. (2018). Epidemiology of canine gastrointestinal helminths in sub‐Saharan Africa. Parasites & Vectors, 11, 1–7. 29458421 10.1186/s13071-018-2688-9 PMC5819185

[vms31258-bib-0021] Chomel, B. B. (2014). Emerging and re‐emerging zoonoses of dogs and cats. Animals, 4, 434–445. 26480316 10.3390/ani4030434 PMC4494318

[vms31258-bib-0022] Croese, J. , Loukas, A. , Opdebeeck, J. , & Prociv, P. (1994). Occult enteric infection by *Ancylostoma caninum*: A previously unrecognized zoonosis. Gastroenterology, 106, 3–12. 8276205 10.1016/s0016-5085(94)93907-1

[vms31258-bib-0097] Cross, J. H. (1992). Intestinal capillariasis. Clinical Microbiology Reviews, 5(2), 120–129. 10.1128/cmr.5.2.120 1576584 PMC358231

[vms31258-bib-0023] Das, S. , Alim, M. A. , Sikder, S. , Gupta, A. D. , & Masuduzzaman, M. (2012). Prevalence and worm load of enteric helminthiasis in stray dogs of Chittagong Metropolitan, Bangladesh. Yüzüncü Yıl Üniversitesi Veteriner Fakültesi Dergisi, 23, 141–145.

[vms31258-bib-0024] Despommier, D. (2003). Toxocariasis: Clinical aspects, epidemiology, medical ecology, and molecular aspects. Clinical Microbiology Reviews, 16, 265–272. 12692098 10.1128/CMR.16.2.265-272.2003 PMC153144

[vms31258-bib-0025] Drake, J. , & Carey, T. (2019). Seasonality and changing prevalence of common canine gastrointestinal nematodes in the USA. Parasites & Vectors, 12, 1–7. 31488192 10.1186/s13071-019-3701-7 PMC6728981

[vms31258-bib-0026] Dubey, J. , Chapman, J. L. , Rosenthal, B. M. , Mense, M. , & Schueler, R. L. (2006). Clinical *Sarcocystis neurona*, *Sarcocystis canis*, *Toxoplasma gondii*, and *Neospora caninum* infections in dogs. Veterinary Parasitology, 137, 36–49. 16458431 10.1016/j.vetpar.2005.12.017

[vms31258-bib-0027] Dubey, J. P. (2003). Review of *Neospora caninum* and neosporosis in animals. The Korean Journal of Parasitology, 41, 1–16. 12666725 10.3347/kjp.2003.41.1.1 PMC2717477

[vms31258-bib-0028] Dunn, J. J. , Columbus, S. T. , Aldeen, W. E. , Davis, M. , & Carroll, K. C. (2002). *Trichuris vulpis* recovered from a patient with chronic diarrhea and five dogs. Journal of Clinical Microbiology, 40, 2703–2704. 12089315 10.1128/JCM.40.7.2703-2704.2002 PMC120537

[vms31258-bib-0029] Eastburn, R. L. , Fritsche, T. R. , & Terhune, C. A., Jr. (1987). Human intestinal infection with *Nanophyetus salmincola* from salmonid fishes. The American Journal of Tropical Medicine and Hygiene, 36, 586–591. 3578655 10.4269/ajtmh.1987.36.586

[vms31258-bib-0030] Eckert, J. , & Deplazes, P. (2004). Biological, epidemiological, and clinical aspects of echinococcosis, a zoonosis of increasing concern. Clinical Microbiology Reviews, 17, 107–135. 14726458 10.1128/CMR.17.1.107-135.2004 PMC321468

[vms31258-bib-0031] Ezeamama, A. E. , McGarvey, S. T. , Acosta, L. P. , Zierler, S. , Manalo, D. L. , Wu, H.‐W. , Kurtis, J. D. , Mor, V. , Olveda, R. M. , & Friedman, J. F. (2008). The synergistic effect of concomitant schistosomiasis, hookworm, and trichuris infections on children's anemia burden. PLoS Neglected Tropical Diseases, 2, e245. 18523547 10.1371/journal.pntd.0000245 PMC2390851

[vms31258-bib-0032] Fang, F. , Li, J. , Huang, T. , Guillot, J. , & Huang, W. (2015). Zoonotic helminths parasites in the digestive tract of feral dogs and cats in Guangxi, China. BMC Veterinary Research, 11, 1–5. 26276147 10.1186/s12917-015-0521-7 PMC4537577

[vms31258-bib-0033] Fayer, R. , Esposito, D. H. , & Dubey, J. P. (2015). Human infections with *Sarcocystis* species. Clinical Microbiology Reviews, 28, 295–311. 25715644 10.1128/CMR.00113-14 PMC4402950

[vms31258-bib-0034] Fleming, M. W. (1997). Cortisol as an indicator of severity of parasitic infections of *Haemonchus contortus* in lambs (*Ovis aries*). Comparative Biochemistry and Physiology Part B: Biochemistry and Molecular Biology, 116, 41–44. 10.1016/s0305-0491(96)00157-5 9080661

[vms31258-bib-0035] Fong, I. (2017). Animals and mechanisms of disease transmission. Emerging Zoonoses, 2017, 15–38.

[vms31258-bib-0036] Freedman, A. H. , & Wayne, R. K. (2017). Deciphering the origin of dogs: From fossils to genomes. Annual Review of Animal Biosciences, 5, 281–307. 27912242 10.1146/annurev-animal-022114-110937

[vms31258-bib-0037] Fritsche, T. R. , Eastburn, R. L. , Wiggins, L. H. , & Terhune, C. A., Jr. (1989). Praziquantel for treatment of human *Nanophyetus salmincola* (*Troglotrema salmincola*) infection. Journal of Infectious Diseases, 160, 896–899. 2809261 10.1093/infdis/160.5.896

[vms31258-bib-0038] Ghasemzadeh, I. , & Namazi, S. (2015). Review of bacterial and viral zoonotic infections transmitted by dogs. Journal of Medicine and Life, 8, 1. PMC5319273 28316698

[vms31258-bib-0039] Ghimire, T. , Adhikari, R. , & Bhattarai, N. (2021). Diversity and prevalence of Eimeria species in goats of Nepal. Journal of the Hellenic Veterinary Medical Society, 72, 3299–3306.

[vms31258-bib-0040] Gibson, A. K. , Raverty, S. , Lambourn, D. M. , Huggins, J. , Magargal, S. L. , & Grigg, M. E. (2011). Polyparasitism is associated with increased disease severity in Toxoplasma gondii‐infected marine sentinel species. PLoS Neglected Tropical Diseases, 5, e1142. 21629726 10.1371/journal.pntd.0001142 PMC3101184

[vms31258-bib-0042] Gutema, F. D. , Yohannes, G. W. , Abdi, R. D. , Abuna, F. , Ayana, D. , Waktole, H. , Amenu, K. , Hiko, A. , & Agga, G. E. (2021). *Dipylidium caninum* infection in dogs and humans in Bishoftu Town, Ethiopia. Diseases, 9, 1. 10.3390/diseases9010001 PMC7839016 33374931

[vms31258-bib-0043] Heukelbach, J. , Jackson, A. , Ariza, L. , & Feldmeier, H. (2008). Prevalence and risk factors of hookworm‐related cutaneous larva migrans in a rural community in Brazil. Annals of Tropical Medicine & Parasitology, 102, 53–61. 18186978 10.1179/136485908X252205

[vms31258-bib-0044] Hoarau, A. O. , Mavingui, P. , & Lebarbenchon, C. (2020). Coinfections in wildlife: Focus on a neglected aspect of infectious disease epidemiology. PLoS Pathogens, 16, e1008790. 32881983 10.1371/journal.ppat.1008790 PMC7470396

[vms31258-bib-0045] Idrissi, H. , Khatat, S. E. H. , Duchateau, L. , Kachani, M. , Daminet, S. , El Asatey, S. , Tazi, N. , Azrib, R. , & Sahibi, H. (2022). Prevalence, risk factors and zoonotic potential of intestinal parasites in dogs from four locations in Morocco. Veterinary Parasitology: Regional Studies and Reports, 34, 100775. 36041810 10.1016/j.vprsr.2022.100775

[vms31258-bib-0046] Igore, K. , Payne, V. , Nadia, N. , & Cedric, Y. (2020). Risk factors associated with prevalence and intensity of gastrointestinal parasitic infections within households in Tonga Sub‐Division, West Region, Cameroon. Journal of Infectious Diseases and Epidemiology, 6, 123.

[vms31258-bib-0047] Jensen, K. D. , Camejo, A. , Melo, M. B. , Cordeiro, C. , Julien, L. , Grotenbreg, G. M. , Frickel, E.‐M. , Ploegh, H. L. , Young, L. , & Saeij, J. P. J. (2015). Toxoplasma gondii superinfection and virulence during secondary infection correlate with the exact ROP5/ROP18 allelic combination. MBio, 6, e02280. 25714710 10.1128/mBio.02280-14 PMC4358003

[vms31258-bib-0048] Jian, F. , Qi, M. , He, X. , Wang, R. , Zhang, S. , Dong, H. , & Zhang, L. (2014). Occurrence and molecular characterization of Cryptosporidium in dogs in Henan Province, China. BMC Veterinary Research, 10, 1–4. 24433398 10.1186/1746-6148-10-26 PMC3896686

[vms31258-bib-0049] Jones, O. , Kebede, N. , Kassa, T. , & Tilahun, G. (2011). Prevalence of dog gastrointestinal parasites and risk perception of zoonotic infection by dog owners in Wondo Genet, Southern Ethiopia. Journal of Public Health and Epidemiology, 3, 550–555.

[vms31258-bib-0050] Kagira, J. , & Kanyari, P. (2000). Parasitic diseases as causes of mortality in dogs in Kenya. A retrospective study of 351 cases (1984–1998). Israel Journal of Veterinary Medicine, 56, 11–99.

[vms31258-bib-0051] Lin, M. , Bachman, K. , Cheng, Z. , Daugherty, S. C. , Nagaraj, S. , Sengamalay, N. , Ott, S. , Godinez, A. , Tallon, L. J. , Sadzewicz, L. , Fraser, C. , Dunning Hotopp, J. C. , & Rikihisa, Y. (2017). Analysis of complete genome sequence and major surface antigens of Neorickettsia helminthoeca, causative agent of salmon poisoning disease. Microbial Biotechnology, 10, 933–957. 28585301 10.1111/1751-7915.12731 PMC5481527

[vms31258-bib-0052] Lupo, P. J. , Langer‐Curry, R. C. , Robinson, M. , Okhuysen, P. C. , & Chappell, C. L. (2008). *Cryptosporidium muris* in a Texas canine population. The American Journal of Tropical Medicine and Hygiene, 78, 917–921. 18541769

[vms31258-bib-0053] Macpherson, C. N. (2013). The epidemiology and public health importance of toxocariasis: A zoonosis of global importance. International Journal for Parasitology, 43, 999–1008. 23954435 10.1016/j.ijpara.2013.07.004

[vms31258-bib-0054] Magnaval, J.‐F. , Glickman, L. T. , Dorchies, P. , & Morassin, B. (2001). Highlights of human toxocariasis. The Korean Journal of Parasitology, 39, 1–11. 11301585 10.3347/kjp.2001.39.1.1 PMC2721060

[vms31258-bib-0055] Magnaval, J.‐F. , Michault, A. , Calon, N. , & Charlet, J.‐P. (1994). Epidemiology of human toxocariasis in La Reunion. Transactions of the Royal Society of Tropical Medicine and Hygiene, 88, 531–533. 7992328 10.1016/0035-9203(94)90148-1

[vms31258-bib-0056] Malgor, R. , Oku, Y. , Gallardo, R. , & Yarzábal, L. (1996). High prevalence of *Ancylostoma* spp. infection in dogs, associated with endemic focus of human cutaneous larva migrans, in Tacuarembo, Uruguay. Parasite, 3, 131–134. 8758550 10.1051/parasite/1996032131

[vms31258-bib-0057] Márquez‐Navarro, A. , García‐Bracamontes, G. , Álvarez‐Fernández, B. E. , Ávila‐Caballero, L. P. , Santos‐Aranda, I. , Díaz‐Chiguer, D. L. , Sánchez‐Manzano, R. M. , Rodríguez‐Bataz, E. , & Nogueda‐Torres, B. (2012). *Trichuris vulpis* (Froelich, 1789) infection in a child: A case report. The Korean Journal of Parasitology, 50, 69. 22451737 10.3347/kjp.2012.50.1.69 PMC3309054

[vms31258-bib-0058] Massei, G. , Fooks, A. , Horton, D. L. , Callaby, R. , Sharma, K. , Dhakal, I. , & Dahal, U. (2017). Free‐roaming dogs in Nepal: Demographics, health and public knowledge, attitudes and practices. Zoonoses and Public Health, 64, 29–40. 27334892 10.1111/zph.12280

[vms31258-bib-0059] McCarthy, J. , & Moore, T. A. (2000). Emerging helminth zoonoses. International Journal for Parasitology, 30, 1351–1359. 11113260 10.1016/s0020-7519(00)00122-3

[vms31258-bib-0060] Mendoza Roldan, J. A. , & Otranto, D. (2023). Zoonotic parasites associated with predation by dogs and cats. Parasites & Vectors, 16, 55. 36747243 10.1186/s13071-023-05670-y PMC9901148

[vms31258-bib-0061] Mirdha, B. , Singh, Y. , Samantray, J. , & Mishra, B. (1998). *Trichuris vulpis* infection in slum children. Indian Journal of Gastroenterology: Official Journal of the Indian Society of Gastroenterology, 17, 154–154. 9795508

[vms31258-bib-0062] Morelli, S. , Marruchella, G. , Passarelli, A. , Diakou, A. , Di Cesare, A. , & Colombo, M. (2021). An unusual case of mixed respiratory capillariosis in a dog. Pathogens, 10, 117. 33498766 10.3390/pathogens10020117 PMC7911517

[vms31258-bib-0063] Morgan, U. , Buddle, J. , Armson, A. , Elliot, A. , & Thompson, R. (1999). Molecular and biological characterisation of Cryptosporidium in pigs. Australian Veterinary Journal, 77, 44–47. 10028394 10.1111/j.1751-0813.1999.tb12428.x

[vms31258-bib-0064] Mugala, L. , Siwila, J. , Saasa, N. , & Pandey, G. S. (2018). Prevalence of *Cryptosporidium* spp. oocysts in dogs in Lusaka district of Zambia. Veterinary World, 11, 585. 29915495 10.14202/vetworld.2018.585-589 PMC5993767

[vms31258-bib-0065] Mukaratirwa, S. , & Singh, V. (2010). Prevalence of gastrointestinal parasites of stray dogs impounded by the Society for the Prevention of Cruelty to Animals (SPCA), Durban and Coast, South Africa. Journal of the South African Veterinary Association, 81, 123–125. 21247022 10.4102/jsava.v81i2.124

[vms31258-bib-0066] Ng‐Nguyen, D. , Hii, S. F. , Nguyen, V.‐A. T. , Van Nguyen, T. , Van Nguyen, D. , & Traub, R. J. (2015). Re‐evaluation of the species of hookworms infecting dogs in Central Vietnam. Parasites & Vectors, 8, 1–6. 26216353 10.1186/s13071-015-1015-y PMC4517506

[vms31258-bib-0067] Ngui, R. , Lee, S. , Yap, N. , Tan, T. , Aidil, R. , Chua, K. , Aziz, S. , Sulaiman, W. , Ahmad, A. , Mahmud, R. , & Lian, Y. (2014). Gastrointestinal parasites in rural dogs and cats in Selangor and Pahang states in Peninsular Malaysia. Acta Parasitologica, 59, 737–744. 25236287 10.2478/s11686-014-0306-3

[vms31258-bib-0068] Nijsse, R. , Mughini‐Gras, L. , Wagenaar, J. A. , & Ploeger, H. W. (2016). Recurrent patent infections with *Toxocara canis* in household dogs older than six months: A prospective study. Parasites & Vectors, 9, 1–11. 27716389 10.1186/s13071-016-1816-7 PMC5051026

[vms31258-bib-0069] Otranto, D. , Strube, C. , & Xiao, L. (2021). Zoonotic parasites: The one health challenge. Parasitology Research, 120, 4073–4074. 34142224 10.1007/s00436-021-07221-9 PMC8211557

[vms31258-bib-0070] Pereira, M. , Valério‐Bolas, A. , Saraiva‐Marques, C. , Alexandre‐Pires, G. , Pereira da Fonseca, I. , & Santos‐Gomes, G. (2019). Development of dog immune system: From in uterus to elderly. Veterinary Sciences, 6, 83. 31640234 10.3390/vetsci6040083 PMC6958461

[vms31258-bib-0071] Sangache, V. C. , Vásquez, W. L. , & Moreira, J. M. (2020). Prevalence of intestinal parasites in dogs in the city of San Miguel DE BOLÍVAR (ECUADOR). PalArch's Journal of Archaeology of Egypt/Egyptology, 17, 14487–14494.

[vms31258-bib-0072] Saravanan, M. , Sarma, K. , Mondal, D. , Ranjith Kumar, M. , & Vijayakumar, H. (2016). Concomitant infestation of *Toxocara cati* and *Ancylostoma tubaeforme* in a mongrel cat. Journal of Parasitic Diseases, 40, 205–207. 27065627 10.1007/s12639-014-0451-5 PMC4815833

[vms31258-bib-0073] Satyal, R. , Manandhar, S. , Dhakal, S. , Mahato, B. , Chaulagain, S. , Ghimire, L. , & Pandeya, Y. (2013). Prevalence of gastrointestinal zoonotic helminths in dogs of Kathmandu, Nepal. International Journal of Infection and Microbiology, 2, 91–94.

[vms31258-bib-0074] Scandurra, A. , Alterisio, A. , Di Cosmo, A. , & D'Aniello, B. (2018). Behavioral and perceptual differences between sexes in dogs: An overview. Animals, 8, 151. 30142932 10.3390/ani8090151 PMC6162565

[vms31258-bib-0075] Schantz, P. M. (2007). Zoonotic parasitic infections contracted from dogs and cats: How frequent are they? Division of Parasitic Diseases, National Center for Zoonotic, Vectorborne and Enteric Diseases, Centers for Disease Control and Prevention, Aatlanta, GAA. https://www.dvm360.com/view/zoonotic-parasitic-infections-contracted-dogs-and-cats-how-frequent-are-they

[vms31258-bib-0076] Shuster, S. M. , & Wade, M. J. (2019). Mating systems and strategies. Princeton University Press.

[vms31258-bib-0077] Singh, A. , Cunningham, E. T. , & Stewart, J. M. (2007). Detection and treatment of ocular toxocariasis. Review of Ophthalmology, 14, 55–58.

[vms31258-bib-0078] Slapeta, J. , Koudela, B. , Votýpka, J. , Modrý, D. , Horejs, R. , & Lukes, J. (2002). Coprodiagnosis of *Hammondia heydorni* in dogs by PCR based amplification of ITS 1 rRNA: Differentiation from morphologically indistinguishable oocysts of *Neospora caninum* . The Veterinary Journal, 163, 147–154. 12093189 10.1053/tvjl.2001.0599

[vms31258-bib-0079] Sokhna, C. , Le Hesran, J.‐Y. , Mbaye, P. A. , Akiana, J. , Camara, P. , Diop, M. , Ly, A. , & Druilhe, P. (2004). Increase of malaria attacks among children presenting concomitant infection by *Schistosoma mansoni* in Senegal. Malaria Journal, 3, 1–5. 15544703 10.1186/1475-2875-3-43 PMC538284

[vms31258-bib-0080] Soulsby, E. J. (2012). Helminths, arthropods, and protozoa of domesticated animals (7th ed.). Affiliated East‐West Press Private Limited,.

[vms31258-bib-0081] Sowemimo, O. A. (2009). The prevalence and intensity of gastrointestinal parasites of dogs in Ile‐Ife, Nigeria. Journal of Helminthology, 83, 27–31. 18838021 10.1017/S0022149X08067229

[vms31258-bib-0082] Sudan, V. , Jaiswal, A. K. , Shanker, D. , Kanojiya, D. , & Sachan, A. (2015). Prevalence of endoparasitic infections of non descript dogs in Mathura, Uttar Pradesh. Journal of Parasitic Diseases, 39, 491–494. 26345058 10.1007/s12639-013-0383-5 PMC4554565

[vms31258-bib-0083] Sukupayo, P. R. , & Tamang, S. (2023). Prevalence of Zoonotic Gastrointestinal Helminth Parasite among Dogs in Suryabinayak, Nepal. Veterinary Medicine International, 2023, 3624593. 37287959 10.1155/2023/3624593 PMC10243950

[vms31258-bib-0084] Sundman, A.‐S. , Van Poucke, E. , Svensson Holm, A.‐C. , Faresjö, Å. , Theodorsson, E. , Jensen, P. , & Roth, L. S. V. (2019). Long‐term stress levels are synchronized in dogs and their owners. Scientific Reports, 9, 1–7. 31171798 10.1038/s41598-019-43851-x PMC6554395

[vms31258-bib-0085] Trasviña‐Muñoz, E. , López‐Valencia, G. , Monge‐Navarro, F. J. , Herrera‐Ramírez, J. C. , Haro, P. , Gómez‐Gómez, S. D. , Mercado‐Rodríguez, J. A. , Flores‐Dueñas, C. A. , Cueto‐Gonzalez, S. A. , & Burquez‐Escobedo, M. (2020). Detection of intestinal parasites in stray dogs from a farming and cattle region of Northwestern Mexico. Pathogens, 9, 516. 32605146 10.3390/pathogens9070516 PMC7400657

[vms31258-bib-0086] Traub, R. J. , Pednekar, R. P. , Cuttell, L. , Porter, R. B. , Rani, P. A. A. M. , & Gatne, M. L. (2014). The prevalence and distribution of gastrointestinal parasites of stray and refuge dogs in four locations in India. Veterinary Parasitology, 205, 233–238. 25139393 10.1016/j.vetpar.2014.06.037

[vms31258-bib-0087] Traub, R. J. , Robertson, I. D. , Irwin, P. , Mencke, N. , & Thompson, R. A. (2002). The role of dogs in transmission of gastrointestinal parasites in a remote tea‐growing community in northeastern India. American Journal of Tropical Medicine and Hygiene, 67, 539–545. 12479559 10.4269/ajtmh.2002.67.539

[vms31258-bib-0088] Tun, S. , Ithoi, I. , Mahmud, R. , Samsudin, N. I. , Kek Heng, C. , & Ling, L. Y. (2015). Detection of helminth eggs and identification of hookworm species in stray cats, dogs and soil from Klang Valley, Malaysia. PLoS One, 10, e0142231. 26671680 10.1371/journal.pone.0142231 PMC4682862

[vms31258-bib-0089] Vanacore, C. B. (2022). Dog. Encyclopedia Britannica. https://www.britannica.com/animal/dog

[vms31258-bib-0090] Vaumourin, E. , Vourc'h, G. , Gasqui, P. , & Vayssier‐Taussat, M. (2015). The importance of multiparasitism: Examining the consequences of co‐infections for human and animal health. Parasites & Vectors, 8, 1–13. 26482351 10.1186/s13071-015-1167-9 PMC4617890

[vms31258-bib-0091] Wakelin, D. (1984). How animals control parasitic infections In. Immunity to Parasites, 1, 93–117.

[vms31258-bib-0092] Wells, K. , Gibson, D. I. , Clark, N. J. , Ribas, A. , Morand, S. , & McCallum, H. I. (2018). Global spread of helminth parasites at the human–domestic animal–wildlife interface. Global Change Biology, 24, 3254–3265. 29436086 10.1111/gcb.14064

[vms31258-bib-0093] Wirant, S. C. , & McGuire, B. (2004). Urinary behavior of female domestic dogs (*Canis familiaris*): Influence of reproductive status, location, and age. Applied Animal Behaviour Science, 85, 335–348.

[vms31258-bib-0094] Yadav, K. , & Shrestha, B. (2017). Prevalence of zoonotic gastrointestional helminth parasites in pet and stray dogs of Rupandehi District, Nepal. Microbiology & Infectious Diseases, 1, 1–7.

[vms31258-bib-0095] Zajac, A. M. , Conboy, G. A. , Little, S. E. , & Reichard, M. V. (2021). Veterinary clinical parasitology (9th ed.). Wiley‐Blackwell.

